# Respiratory pathogens during the COVID-19 pandemic: Alterations in detection and seasonality in Nashville, Tennessee

**DOI:** 10.1371/journal.pone.0270469

**Published:** 2022-08-03

**Authors:** Zaid Haddadin, Andrew J. Spieker, Herdi Rahman, Danielle A. Rankin, Rana Talj, Ahmad Yanis, Justin Z. Amarin, Jonathan Schmitz, James Chappell, Natasha B. Halasa

**Affiliations:** 1 Department of Pediatrics, Vanderbilt University Medical Center, Nashville, TN, United States of America; 2 Department of Biostatistics, Vanderbilt University Medical Center, Nashville, TN, United States of America; 3 Vanderbilt Epidemiology PhD Program, Vanderbilt University School of Medicine, Nashville, TN, United States of America; 4 Departments of Pathology, Microbiology and Immunology, Vanderbilt University Medical Center, Nashville, TN, United States of America; Carol Davila University of Medicine and Pharmacy: Universitatea de Medicina si Farmacie Carol Davila, ROMANIA

## Abstract

Shortly after the implementation of community mitigation measures in response to severe acute respiratory syndrome coronavirus-2 (SARS-CoV-2), sharp declines in respiratory syncytial virus and influenza circulation were noted; post-mitigation circulation of other respiratory pathogens has gone unexplored. We retrospectively analyzed all records of a provider-ordered multiplex test between April 1, 2018, and July 31, 2021, in Nashville, Tennessee, and we noted disrupted historical seasonal patterns for common respiratory pathogens during the SARS-CoV-2 pandemic.

## Introduction

A decrease in medically attended acute respiratory illness (ARI) due to pathogens other than severe acute respiratory syndrome coronavirus 2 (SARS-CoV-2) has been reported worldwide during the coronavirus disease 2019 (COVID-19) pandemic [1–3]. For instance, we previously showed that the circulation of respiratory syncytial virus (RSV) and influenza ceased abruptly in March 2020 shortly after the implementation of community mitigation measures to lessen the spread of SARS-CoV-2 across seven cities in the United States [4]. This study of children under 18 years with ARI noted an average of 10.6 fewer eligible ARI cases/week per site and 63.9% and 45.8% lower odds of testing positive for RSV and influenza, respectively, during the 2020 community mitigation period compared to the 2016–2019 period. Nashville was one of the study sites, and on March 23, 2020, the Metro Public Health Department in Nashville, Tennessee issued a stay-at-home order for non-essential workers in Davidson County [5]; and by March 31, 2020, the Tennessee governor issued a state-wide mandate [6]. The following phase implementations for Nashville included no restrictions (pre-pandemic April 2018–March 22, 2020); stay-at-home order (March 23, 2020–May 10, 2020); Phase I (May 11, 2020-May 24, 2020); Phase II (May 25, 2020-June 28, 2020); Phase III (June 29, 2020-July 1, 2020); Phase II (July 2, 2020-September 30, 2020); then back to Phase III (October 1, 2020-July 31, 2021) [5–14]. SARS-CoV-2 vaccination began in January 2021 [15].

The sharp decline in respiratory pathogen circulation may be attributable to a variety of factors, including mitigation measures, refocused clinical testing and epidemiologic surveillance, altered healthcare-seeking behaviors, and SARS-CoV-2 biological interference with infection by other common respiratory pathogens [16]. While most studies have focused on RSV and influenza circulation amidst the pandemic wave [1, 4], scant information exists on the detection of other significant ARI pathogens.

Therefore, we comprehensively evaluated the absolute and relative detection frequencies of common respiratory pathogens in a clinical setting during the COVID-19 pandemic and compared these results to annual patterns of pathogen detection during the two preceding pre-pandemic years at a large academic medical center in Nashville, TN. Furthermore, we documented respiratory pathogen circulation as restrictions were loosened and the different phases were implemented over time.

## Methods

This retrospective study included all children and adults who had a provider-ordered multiplex BioFire® FilmArray Respiratory Pathogen Panel (RPP) 2.0 test between April 1, 2018, and July 31, 2021, or SARS-CoV-2 testing between March 9, 2020, and July 31, 2021, at Vanderbilt University Medical Center (VUMC) in Nashville, Tennessee. All tests ordered by a VUMC provider were included in these analyses, regardless of the reason they were ordered. The BioFire® RPP 2.0 simultaneously tests for common respiratory viruses (human adenovirus [HAdV]; human coronaviruses HKU1, NL63, 229E, and OC43 [HCoVs]; influenza A, A/H1, A/H3, A/H1-2009, and B [Flu]; human metapneumovirus [HMPV]; parainfluenza virus [PIV]; human rhinovirus/enterovirus [HRV/EV]; and RSV) and bacteria (*Bordetella parapertussis*, *Bordetella pertussis*, *Chlamydia [Chlamydophila] pneumoniae*, and *Mycoplasma pneumoniae*). Molecular testing for SARS-CoV-2 is performed separately as a single-plex polymerase chain reaction test. The results of these laboratory tests were obtained from the clinical molecular diagnostic laboratory records. The specimens were collected as either anterior nasal and/or nasopharyngeal samples. The study protocol was approved by the Institutional Review Board at Vanderbilt University.

### Statistical analyses

The absolute and relative frequency of detection was aggregated by month for each pathogen. Time periods were divided into the following: pre-pandemic (April 2018–Feb 2019; March 2019–Feb 2020), March 2020 as the transitional month, and pandemic (April 2020–Feb 2021, March 2021–July 2021). For each pathogen detected by BioFire®, we used generalized estimating equations with a logistic link function to obtain month-specific odds ratios and 95% confidence intervals comparing the odds of a positive test result for each month over the SARS-CoV-2 pandemic period (March 2020–July 2021) to the same respective month during prior non-pandemic years. We adjusted for age and used a working-independence correlation structure with cluster-based robust standard errors to account for repeated tests on subjects. For influenza, RSV, and HRV/EV, we additionally conducted a subgroup analysis within the following four age groups: <5, 5–17, 18–64, and ≥65 years.

## Results

From April 1, 2018, to July 31, 2021, 36,226 BioFire® tests were performed on 26,445 unique subjects; 13,075 (36.1%) of the tests were positive for at least one pathogen. The three most commonly detected viruses were HRV/EV, RSV, and PIV (with relative detection frequencies of 19.1%, 5.7%, and 4.5%, respectively).

**Fig 1:**
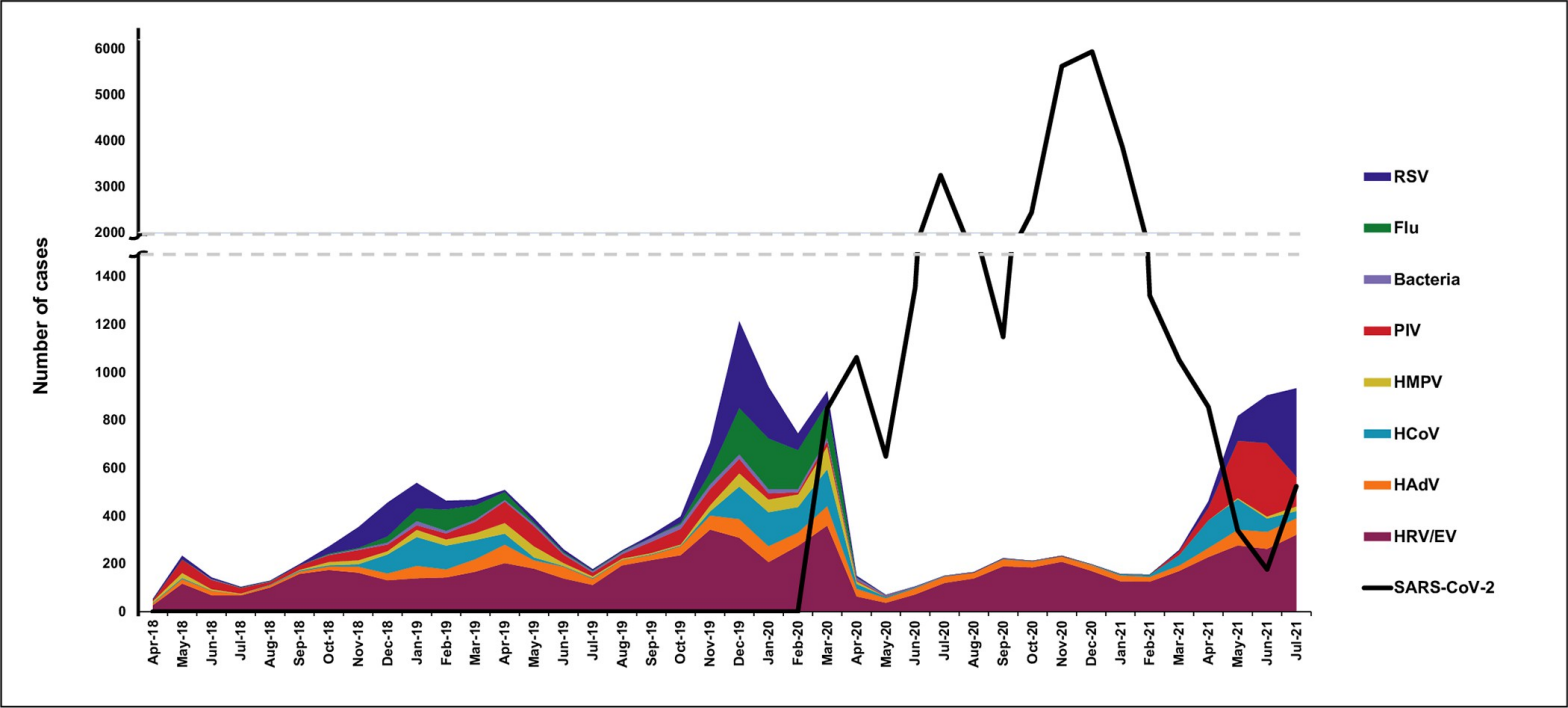
Frequency of respiratory pathogens from April 1, 2018, through July 31, 2021. Abbreviations: RSV, respiratory syncytial virus; Flu, influenza; PIV, parainfluenza virus; HMPV, human metapneumovirus; HCoV, human coronaviruses; HAdV, human adenovirus; HRV/EV, human rhinovirus/enterovirus; SARS-CoV-2, severe acute respiratory syndrome coronavirus 2.

**Fig 2:**
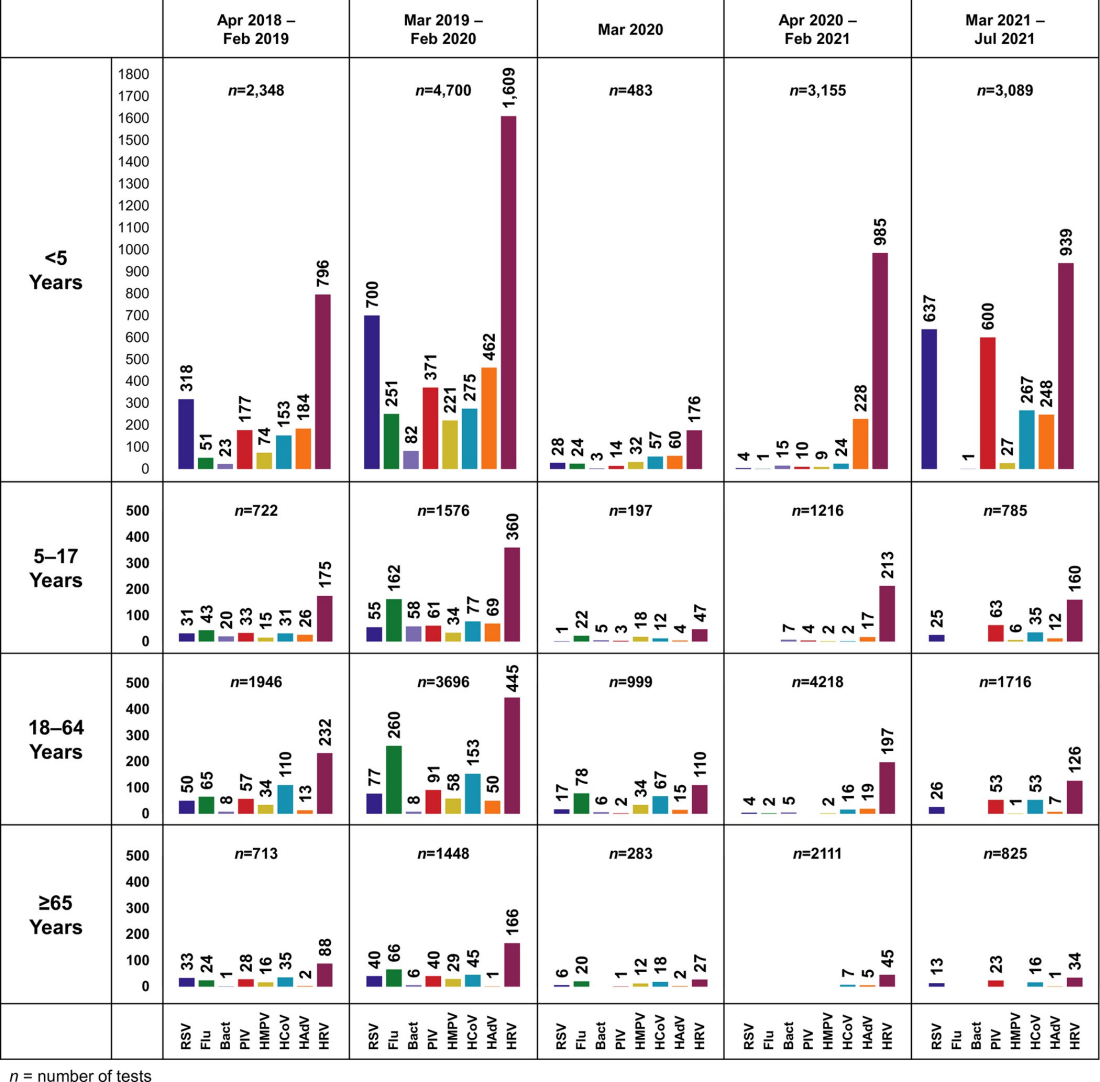
Frequency of respiratory pathogens from April 1, 2018, through July 31, 2021, stratified by age groups and by following the periods: Pre-pandemic (April 2019-Feb 2019; March 2019-Feb 2020), March 2020 as the transitional month, and pandemic period (April 2020-Feb 2021, March 2021-July 2021). Abbreviations: RSV, respiratory syncytial virus; Flu, influenza; PIV, parainfluenza virus; HMPV, human metapneumovirus; HCoV, human coronaviruses; HAdV, human adenovirus; HRV/EV, human rhinovirus/enterovirus.

In total, 17,149 Biofire® tests were performed in the pre-pandemic period, 1,962 were performed in March 2020, and 17,115 were performed during the pandemic period (**Fig 1, Table 1**). Most tests in both the pre-pandemic and pandemic period were performed in children <5 years (41.1% and 36.5%) and 18–64 years (32.9% and 34.7%), respectively (**Fig 2A and 2B).**

**Table 1:** Total number of tests stratified by time period and respiratory pathogens.

	Apr 2018–Feb 2019	Mar 2019–Feb 2020	Mar 2020*	Apr 2020–Feb 2021	Mar 2021–Jul 2021
	**Number of Tests**				
**Pathogen**	***N* = 5,729**	***N* = 11,420**	***N* = 1,962**	***N* = 10,700**	***N* = 6,415**
RSV	432 (7.5)	872 (7.6)	52 (2.7)	8 (0.1)	701 (10.9)
Influenza	183 (3.2)	739 (6.5)	144 (7.3)	3 (0.0)	0 (0.0)
Bacteria	52 (0.9)	154 (1.3)	14 (0.7)	27 (0.3)	1 (0.0)
PIV	295 (5.1)	563 (4.9)	20 (1.0)	14 (0.1)	739 (11.5)
HMPV	139 (2.4)	342 (3.0)	96 (4.9)	13 (0.1)	34 (0.5)
HCoV	329 (5.7)	550 (4.8)	154 (7.8)	49 (0.5)	371 (5.8)
HAdV	225 (3.9)	582 (5.1)	81 (4.1)	269 (2.5)	268 (4.2)
HRV/EV	1291 (22.5)	2580 (22.6)	360 (18.3)	1440 (13.5)	1259 (19.6)
	**Number of Tests**				
	** **	** **	***N* = 10,177**	***N* = 255,644**	***N* = 76,753**
SARS-CoV-2			849 (8.3)	28251 (11.1)	2947 (3.9)

*March 2020 was used as the referent date for the pandemic because Tennessee’s stay-at-home order was issued at the end of the month. **Abbreviations:** RSV, respiratory syncytial virus; Flu, influenza; PIV, parainfluenza virus; HMPV, human metapneumovirus; HCoV, human coronaviruses; HAdV, human adenovirus; HRV/EV, human rhinovirus/enterovirus; SARS-CoV-2, severe acute respiratory syndrome coronavirus 2.

**Fig 1** depicts the frequency of detection for each respiratory pathogen over time, with the first detection of SARS-CoV-2 occurring on March 9, 2020. Before the start of the SARS-CoV-2 pandemic, RSV, HCoVs, and HMPV were most commonly detected from October through April, with early- to late-winter peaks. HRV/EV, HAdV, and PIV were detected year-round with variable peaks. Following community mitigation, there was an abrupt decline in the absolute detection frequencies of all pathogens (**Fig 1**). This was consistent when stratified by all age groups (**Fig 2A and Fig 2B**).

HRV/EV and HAdV circulated less during the initial phase of the pandemic but then continued to circulate throughout the pandemic. In stark contrast, frequencies of detection for influenza (except only two specimens in April 2020 and one specimen in December 2020) and HMPV (one detected specimen in each October and December 2020) were markedly reduced; whereas, new detections of RSV, HCoVs, and PIV were noted again starting in March 2021 (**Fig 1**).

**Fig 3:**
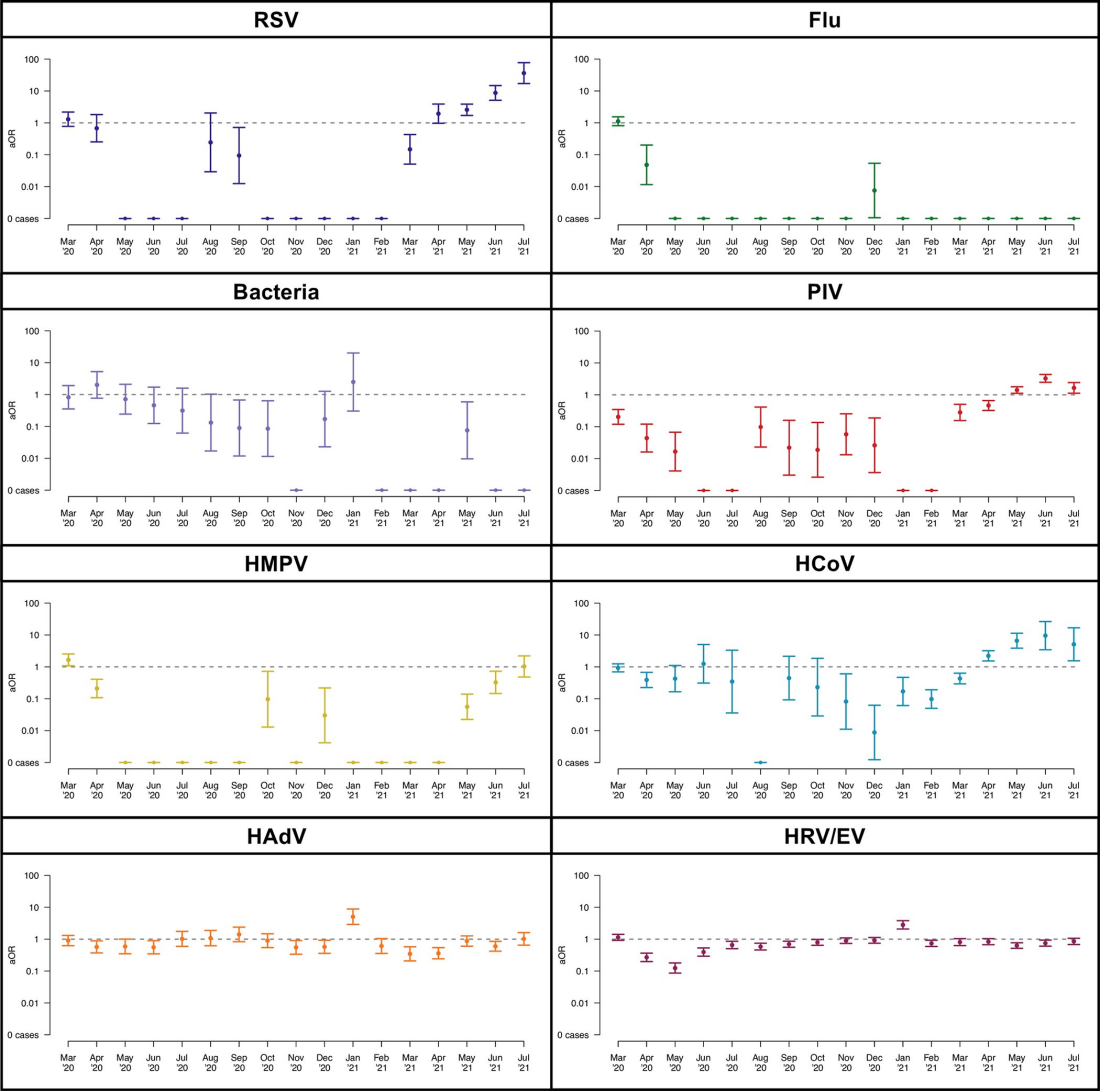
Virus-specific odds ratios, comparing the odds of a positive test result, for each month over the SARS-CoV-2 pandemic period (March 2020 through July 2021) to the same respective months during prior non-pandemic years. Abbreviations: RSV, respiratory syncytial virus; Flu, influenza; PIV, parainfluenza virus; HMPV, human metapneumovirus; HCoV, human coronaviruses; HAdV, human adenovirus; HRV/EV, human rhinovirus/enterovirus.

We found that each tested respiratory pathogen showed statistically significant reductions in the odds of detection in the pandemic period compared to the pre-pandemic period at variable points throughout the pandemic period (**Fig 3**). The relative odds of influenza detection sharply declined in April 2021 (aOR, 0.05; 95% CI, 0.01–0.20; *p*<0.001), with no cases detected for the remainder of the study period except December (one case). RSV showed a substantially lower odds of detection throughout most of the period from May 2020 to March 2021, but with consistently and markedly higher odds of detection in May 2021 (aOR, 2.58; 95% CI, 1.71–3.88; *p*<0.001) through July 2021 (aOR, 36.4; 95% CI, 17.1–77.6; *p*<0.001). Detection of PIV and HMPV showed similar patterns to that of RSV (**Fig 3**). The odds ratios for HCoV have been variable throughout the pandemic achieving significantly lower detection frequencies from November 2020 (aOR, 0.08; 95% CI, 0.01–0.60; *p* = 0.014) through March 2021 (aOR, 0.43; 95% CI, 0.29–0.63; *p*<0.001), but higher detection frequencies from April 2021 (aOR, 2.22, 95% CI, 1.53–3.21; *p*<0.001) through July 2021 (aOR, 5.10; 95% CI, 1.55–16.81; *p* = 0.007).

The relative odds of testing positive for respiratory bacteria showed a delayed but gradual decline with no cases detected between February and July 2021, except May 2021 (aOR, 0.08; 95% CI, 0.01–0.59; *p* = 0.014). HRV/EV demonstrated a brief period of a substantial decline in odds of detection compared to previous years starting in April 2020 (aOR, 0.27; 95% CI, 0.20–0.37; *p*<0.001), and achieving the lowest odds ratio in May 2020 (aOR, 0.12; 95% CI, 0.09–0.18; *p*<0.001), but the relative reduction in circulation during the pandemic period has otherwise been far less pronounced.

Further, there have been sporadic periods of reduced relative odds of detection of HAdV, including from April 2020 (aOR, 0.57; 95% CI, 0.37–0.89; *p*<0.012) to June 2020 (aOR, 0.56; 95% CI, 0.35–0.90; *p* = 0.016), and from March 2021 (aOR, 0.35; 95% CI, 0.21–0.58; *p*<0.001) to April 2021 (aOR, 0.36; 95% CI, 0.24–0.54; *p*<0.001). Both HRV/EV (aOR, 2.81; 95% CI, 2.08–3.81; *p*<0.001) and HAdV (aOR, 5.06; 95% CI, 2.90–8.84; *p*<0.001) had higher relative odds of detection in January 2021 as compared to the pre-pandemic period.

**Fig 4:**
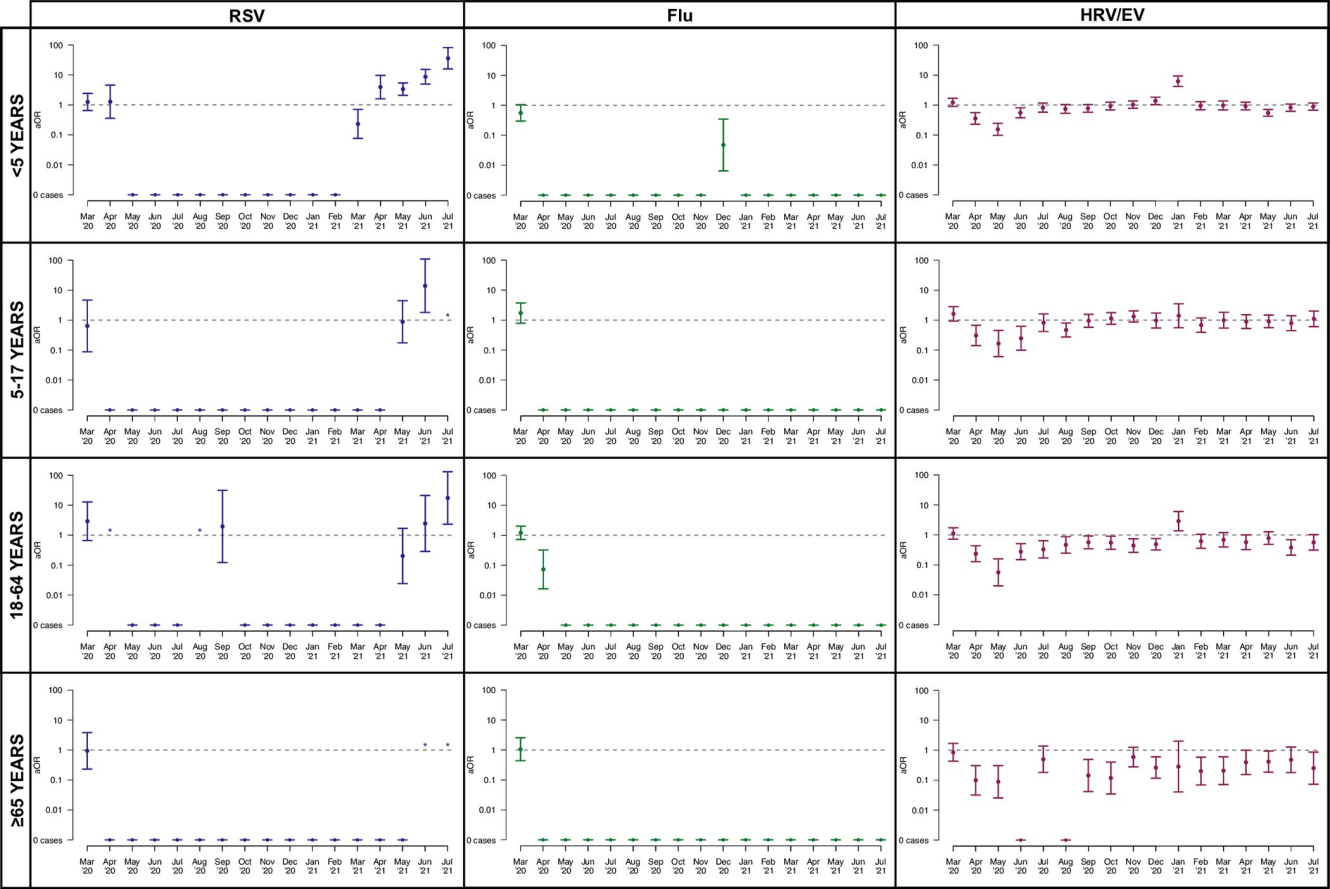
Odds ratios, comparing age group-specific odds of a positive test result for each Flu, RSV, and HRV/EV, for each month over the SARS-CoV-2 pandemic period (March 2020 through July 2021) to the same respective months during prior non-pandemic years. Months in which there were zero cases during the available non-pandemic period are denoted by an asterisk.

**Fig 4** presents corresponding odds ratios comparing the odds of detection for RSV, influenza, and HRV/EV by month during the pandemic period (relative to the pre-pandemic period), stratified by age group. Overall, the patterns within age subgroups mirror those observed within the overall cohort.

## Discussion

Compared to the pre-pandemic period, we noted a marked decline in the clinical laboratory detection of all common respiratory pathogens shortly after community mitigation measures were implemented in response to the COVID-19 pandemic, consistent with other studies worldwide [2, 4, 16, 17]. This pattern can be plausibly explained by 1) community mitigation practices such as social distancing, universal mask-wearing, avoiding crowds or large social gatherings, and closures of schools and non-essential businesses and 2) decreases in healthcare visits during the pandemic, driven by modified practices and priorities to mitigate virus spread and preserve limited resources. In our study, the former explanation better accounts for observed trends given the increased frequency of clinically ordered respiratory pathogen tests during the pandemic compared to prior years, and decreased circulation of agents other than SARS-CoV-2 relative to their respective pre-pandemic levels.

Unique to our study, we demonstrate that circulation and seasonality were not equally or uniformly altered for all pathogens. For instance, the degree of change in HRV/EV and HAdV circulation compared to prior seasons was less pronounced. HRV/EV and HAdV detection declined during the initial stringent community measures implemented in Nashville, TN. However, the circulation of these two viruses was comparable to prior years during the period after which community mitigation measures were loosened. Several factors could explain these observations. First, community mitigation measures may differentially affect the transmission of different respiratory viruses; notably, one study found no evidence that surgical face masks reduce viral shedding of HRV in respiratory droplets or aerosols [18]. Further, HRV and HAdV are non-enveloped viruses, which makes them environmentally stable [17, 19]. A recent study by Murray *et al*. evaluating respiratory pathogen detection on high-touch surfaces at three public elementary schools in Seattle, WA, showed that HRV and HAdV were the most commonly detected viruses on these surfaces [19]. Furthermore, a study in Germany using upper respiratory tract specimens from 3,580 patients with ARI also noted the HRV resurged to levels equaling those of previous years while multiple other respiratory viruses declined in circulation in association with mask mandates [20]. Thus, these factors may explain why the circulation of these particular viruses was less affected by community mitigation measures compared to other respiratory pathogens evaluated in our study.

Unlike HRV/EV and HAdV, the other pathogens evaluated in our study ceased circulating by April 2020 and remained quiescent during the 2020–21 season, a striking departure from normal circulation patterns. For example, there was little to no influenza, HMPV, or bacterial detection noted from the start of the pandemic until April 2021, and RSV, HCoVs, and PIV detection were negligible until March through July 2021. Notably, this appears to be driven by a recent uptick in cases rather than by a lower rate in prior seasons. The recent reappearance of influenza and RSV has been documented in different regions around the globe [1, 21], which supports the validity of our findings and indicates a set of common behavioral, environmental, and/or biologic principles underlying the phenomenon. Further surveillance is needed to document potentially new seasonal patterns of these viruses.

Our study has some limitations. We evaluated laboratory testing results and did not assess the baseline and clinical characteristics of the patients, except for age, thus limiting the generalizability of our study. In addition, the decision to order an RPP for an individual patient was at the discretion of the provider. However, nonbinding guidance from the institution’s Infection Prevention and Stewardship groups encourages that RPPs be obtained on individuals who are admitted or being evaluated for admission in the emergency department if a respiratory infection is suspected. Conversely, according to Infectious Diseases Society of America guidelines, RPPs are not encouraged in routine outpatient evaluation of immunocompetent individuals. Therefore, the practices of providers are unlikely to have changed considerably before and during the pandemic because these guidelines remained in place throughout. Furthermore, we did not assess co-detection of SARS-CoV-2 and other respiratory pathogens, since we analyzed these tests separately. Study strengths include evaluating pathogen detection among patients of all age groups at a referral hospital and satellite clinics serving a large metropolitan area. Further, using a multi-pathogen molecular respiratory diagnostic panel allowed for broad, simultaneous testing of all common ARI pathogens.

The COVID-19 pandemic and associated non-pharmaceutical interventions appear to have disrupted historical seasonal patterns of respiratory pathogen dynamics. Ongoing active ARI surveillance of trends before, during, and after interventional responses to public health emergencies and pandemics is critical to documenting the relative contributions of classical and emerging pathogens to overlapping disease presentations. This information will help guide the clinical management of patients and facilitate timely implementation of appropriate preventive, diagnostic, and treatment measures, and guide the public health response.

## Supporting information

S1 Data (CSV)
